# A computer vision approach for analyzing label free leukocyte trafficking dynamics on a microvascular mimetic

**DOI:** 10.3389/fimmu.2023.1140395

**Published:** 2023-03-24

**Authors:** S. Danial Ahmad, Mujdat Cetin, Richard E. Waugh, James L. McGrath

**Affiliations:** ^1^ Department of Biomedical Engineering, University of Rochester, Rochester, NY, United States; ^2^ Department of Electrical and Computer Engineering, University of Rochester, Rochester, NY, United States; ^3^ Goergen Institute for Data Science, University of Rochester, Rochester, NY, United States

**Keywords:** neutrophil, state detection, computer vision, machine learning, particle tracking, big data, phase contrast, fMLP

## Abstract

High-content imaging techniques in conjunction with *in vitro* microphysiological systems (MPS) allow for novel explorations of physiological phenomena with a high degree of translational relevance due to the usage of human cell lines. MPS featuring ultrathin and nanoporous silicon nitride membranes (µSiM) have been utilized in the past to facilitate high magnification phase contrast microscopy recordings of leukocyte trafficking events in a living mimetic of the human vascular microenvironment. Notably, the imaging plane can be set directly at the endothelial interface in a µSiM device, resulting in a high-resolution capture of an endothelial cell (EC) and leukocyte coculture reacting to different stimulatory conditions. The abundance of data generated from recording observations at this interface can be used to elucidate disease mechanisms related to vascular barrier dysfunction, such as sepsis. The appearance of leukocytes in these recordings is dynamic, changing in character, location and time. Consequently, conventional image processing techniques are incapable of extracting the spatiotemporal profiles and bulk statistics of numerous leukocytes responding to a disease state, necessitating labor-intensive manual processing, a significant limitation of this approach. Here we describe a machine learning pipeline that uses a semantic segmentation algorithm and classification script that, in combination, is capable of automated and label-free leukocyte trafficking analysis in a coculture mimetic. The developed computational toolset has demonstrable parity with manually tabulated datasets when characterizing leukocyte spatiotemporal behavior, is computationally efficient and capable of managing large imaging datasets in a semi-automated manner.

## 1 Introduction

Vascular barrier dysfunction is associated with multiple diseases such as sepsis (1), Alzheimer’s disease (2), and multiple sclerosis (MS) (3). Sepsis is prominent for being a leading cause of death in intensive care units (4), widely prevalent (1.5 million cases in the US annually (5)), and highly costly to healthcare systems (>$20 billion dollars annually) (6). Additionally, Alzheimer’s disease and MS are both prevalent (roughly 900k new cases per year (7) and 900k current patients (8), respectively) and far more costly to the US healthcare system (>$305 billion dollars (9) versus >$85 billion dollars, respectively). Normal vascular endothelium is characterized by tight barriers and low permeability (10), providing a host with homeostatic fluid balance and selective immune cell trafficking (11). Under excessive inflammation, however, vascular barriers experience dysfunction, including higher vascular wall permeability (12), which advances disease progression. Animal models have demonstrated robust organ damage in areas of excessive leukocyte recruitment (13–15), and survivors of severe sepsis often suffer from cognitive impairments (16, 17) due to a variety of factors including blood brain barrier (BBB) infiltration by blood-borne leukocytes that participate in the escalating inflammatory response (18, 19). Importantly, transmigration can also occur under non-inflammatory conditions in the vasculature. For example, monocytes are known to routinely enter the outer meningeal spaces to monitor cerebrospinal fluid for infection (20) and subsets of CD4^+^ T-Cells preferentially cross the BBB (21) to perform immune surveillance. Thus, dysregulated immune cell trafficking is a characteristic of multiple pathological states yet studying the dynamic interplay between vascular barriers and leukocytes *in vivo* is difficult due to limited imaging techniques and a lack of translational fidelity of animal models.

Recently, the emergence of microphysiological systems (MPS) for *in vitro* tissue models has facilitated the exploration of vascular physiology in a living mimetic of a tissue microenvironment (22, 23). Notably, there is an increasing ability to recreate microscale vascular structures (24) in these systems. This enables MPS as a useful platform for directly investigating human mechanisms of vascular barrier dysfunction for the development of future pharmaceutical interventions (25) through the use of human cell lines. Studies performed with MPS typically involve end-point assays such as immunofluorescence or ELISA, and imaging on vascular MPS is limited by the use of components that interfere with image quality, including optically opaque membranes (26). Most MPS systems are not suitable for studying leukocyte trafficking dynamics as this requires high quality live imaging of the blood/tissue interface.

**Figure 1:**
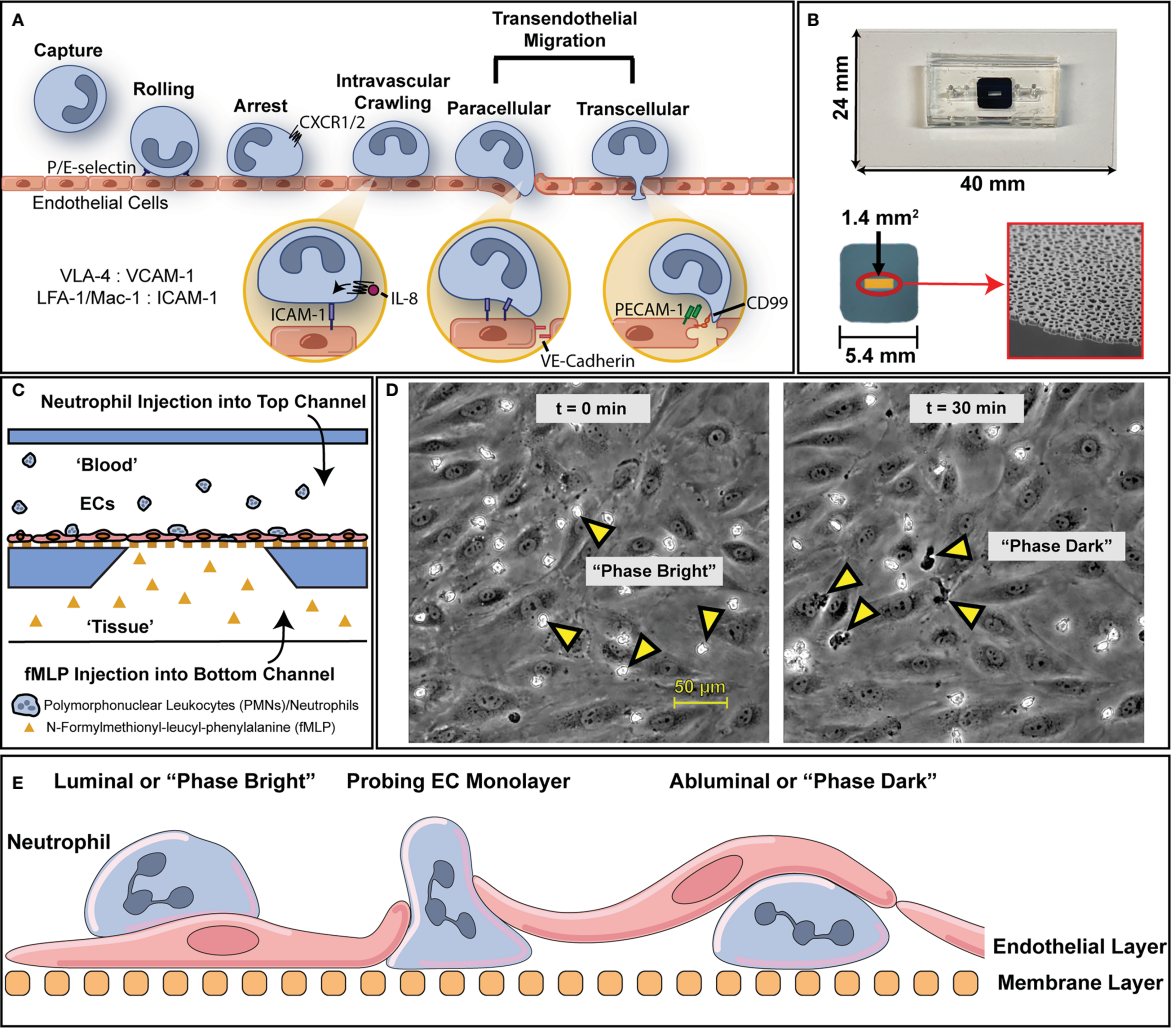
An overview of the leukocyte adhesion cascade and how it is modelled *via* µSiM device. **(A)** To address inflammatory insults, leukocytes engage in an adhesion cascade on the luminal surface of blood vessels that’s mediated through tissue generated chemokine gradients (e.g., IL-8) and surface ligand expression *via* cytokine stimulation (e.g., LFA-1 on PMNs, ICAM-1 on ECs). Through this process, leukocytes are able to transmigrate through endothelium into inflamed tissue where they can then act to clear out local infections. **(B)** To model this behavior and preserve the essential physiology of vascular endothelium, we use microfluidic, silicon membrane-enabled microvascular mimetics (μSiM-MVMs). Layer by layer assembly *via* established protocols results in devices featuring accessible top and bottom microfluidic channels that are separated by an ultra-thin, optically transparent, and highly permeable membrane with nanoscale pores. Devices are built on top of a 24 by 40 mm glass cover slide. The golden window indicates the active porous region of the membrane surface (1.4 mm^2^) which contains nanopores that are viewable *via* scanning electron microscopy, as shown on the bottom right. **(C)** The presence of two channels enables the generation of chemokine gradients to which ECs and leukocytes can respond. For the purposes of this study, devices were seeded with human umbilical vein endothelial cells (HUVECs) and human polymorphonuclear leukocytes (PMNs) were induced to transmigrate with a 10 nM N-Formyl-methionyl-leucyl-phenylalanine (fMLP) gradient. **(D)** Upon PMN introduction, PMNs appear morphologically rounded and “phase bright”, in contrast with the gray contrast of the background ECs. Over the course of 30 minutes, multiple PMNs transmigrate and appear “phase dark”. **(E)** A diagram with nomenclature of PMN transmigration behavior in relation to the microfluidic device. PMNs that are above the endothelium are on the luminal surface or “phase bright” while PMNs that have transmigrated are abluminal or “phase dark”.

In our lab, we have developed the µSiM platform (27) as a modular microfluidic system that offers superior imaging quality for studying neutrophil transmigration in blood vasculature models (**Figure 1A**). The µSiM platform mitigates imaging issues found in conventional MPS platforms as it features optically clear (<100 nm thick) nanoporous silicon nitride (NPN) membranes separating apical and basal compartments of a simple microfluidic device (**Figure 1B**). The membrane enables multiple imaging modalities such as phase-contrast (24), confocal (28), and electron microscopy (29, 30). The µSiM platform facilitates visualization of the vascular wall and has successfully been used to provide high-quality real-time phase contrast video data of polymorphonuclear leukocyte (PMN) or neutrophil trafficking events in a vascular mimetic under inflammatory stimuli (24) (**Figures 1C–E**). Furthermore, ultrathin NPN membranes can be utilized to create cocultures on either side of the membrane, which has been used in a human blood brain barrier (BBB) coculture mimetic for assessing fluorescent particle translocation *via* spinning disc confocal (SDC) microscopy (28). Despite this advancement, analysis of imagery obtained from these studies is typically limited to manual data processing. Given the large image datasets that are generated (31), manual processing is laborious and time-consuming and limits the practical size of data sets that can be obtained. Implementation of automated image analysis will have enormous practical value in terms of the size and completeness of data sets that can be obtained and the avoidance of human bias in data interpretation.

To address the need for automated analysis of high content imagery, semantic segmentation *via* fully convolutional neural networks (CNN) have been developed for pixel level classification of biological images (32, 33). By individually clustering related pixels together for object detection, such machine learning (ML) algorithms can create high contrast maps delineating the spatiotemporal behavior of dynamic leukocytes engaged in trafficking in a µSiM device, which is difficult to do with conventional algorithms (24). The data can then be managed in a semi-automated manner through custom programs and be used to analyze relevant details from an experiment. ML algorithms need to be adapted on a case-by-case basis for any given imagery dataset. They have been used successfully for *in vivo* label-free tracking and imaging of leukocytes in retina (34), providing a basis for automated analysis of large microscopy datasets. Here, we present a computational toolset that leverages machine learning algorithms to facilitate high accuracy monitoring of PMN trafficking and tracking behavior in a vascular mimetic, ultimately minimizing compound errors due to human bias and providing a key step in the development of a high throughput assay.

## 2 Materials and methods

### 2.1 µSiM manufacture

Microphysiological systems featuring microfluidic channels and highly permeable silicon nitride membranes (µSiM flow cells) were manufactured in accordance to protocol as described elsewhere (30). Briefly, 300 µm thick sheets of silicone gasket (Trelleborg Sealing Solutions, Trelleborg, Sweden) and 130 µm thick sheets of pressure sensitive adhesive (3M, Maplewood, MN) were precision cut and assembled layer by layer into µSiM devices using an irreversible bonding step *via* UV-ozone treatment (15 minutes) and thermal incubation (70°C for 2 hours). The devices featured a layer containing a nanoporous silicon nitride membrane (SiMPore Inc., West Henrietta, NY) that contains a freestanding window that is <100 nm thick, with an average pore diameter of 60 nm and overall porosity of ~15%.

### 2.2 Cell culture (HUVECs)

Human umbilical vein endothelial cells (HUVECs) were purchased from a biological supply vendor (Vec Technologies Inc, Rennselaer, NY) and expanded in plastic tissue culture flasks (T25) containing MCDB-131 complete media (Vec Technologies Inc., Rennselaer, NY). HUVECs were maintained in standard cell culture incubation settings (5% CO_2_, 37°C) and used for device seeding between passages 2 and 6. Prior to seeding HUVECs, µSiM devices were first autoclaved and subsequently exposed to UV in a cell culture hood for 15 minutes. Post sterilization, the top channels of the µSiM devices were coated with 5 µg/cm (2) fibronectin (FN-1918, R&D Systems) for one hour at room temperature to facilitate endothelial cell adhesion. HUVECs were seeded into the fibronectin coated top channels at 40,000 cells/cm (2) and maintained in a cell culture incubator (5% CO_2_, 37°C) for 24 hours in static conditions prior to experimentation. Experimental groups were split into positive and negative control studies. In negative control studies devices were maintained with MCDB-131 complete media, whereas in positive control studies 10 nM of N-formyl-met-leu-phe (fMLP) was added to the media in the bottom channel (abluminal side) of the device to serve as a PMN chemoattractant.

### 2.3 PMN isolation

Human PMNs were isolated from whole blood obtained from consenting donors following a protocol that has been approved by the University of Rochester Institutional Review Board (IRB). Briefly, whole blood was drawn into 10 mL sodium heparin coated tubes (B.D., Franklin Lakes, NJ) from healthy donors and cooled to room temperature over 20 minutes. Upon cooling, whole blood was layered on top of a density gradient ‘1-Step Polymorphs’ solution (Accurate Chemical & Scientific Co., Westbury, NY) and separated following manufacturer protocols (500g, 30 minutes, 20°C). All layers except for the PMN rich layer were discarded. The PMN rich layer was diluted and washed in a buffer consisting of Hank’s balanced salt solution (calcium and magnesium free), 10 mM of 4-(2-Hydroxyethyl) piperazine-1-ethanesulfonic acid (HEPES) sodium salt, and 5 mg/mL bovine serum albumin. PMNs were pelleted (350g, 10 minutes, 20°C) and resuspended twice in wash buffer before being depleted of red blood cells *via* hypotonic lysis. Post lysis, the PMNs were suspended and washed once before being deposited in a 1.5 mL Eppendorf conical filled with 1 mL of wash buffer. Fully isolated PMNs were left on a rotating stand to prevent settling and used for experiments within 3 hours post isolation to minimize changes in cell properties.

### 2.4 Microscope studies

Isolated PMNs were suspended in MCDB-131 complete media and introduced into the top channel of sterile µSiM devices with confluent HUVEC monolayers at a seeding density of 3 million PMNs/mL, which matches healthy physiological PMN counts in the human body (35). This density was also chosen to limit excessive population counts in a field of view, ensuring a higher accuracy for the automated workflow. In negative control studies, devices were flushed with sterile media in both channels prior to PMN introduction, while in positive control studies, fMLP rich media was infused into the bottom channel of the device to serve as a potent PMN chemoattractant. After introducing PMN’s *via* pipette injection, devices were placed inside an incubation stage (37°C) coupled with an inverted microscope (Nikon Ti2E, Nikon Corporation, Tokyo, Japan) and recorded in a phase contrast imaging modality (0.25 Hz) for 30 minutes *via* Zyla sCMOS camera (Andor Technology, Belfast, UK) and 40x long working distance lens (NA 0.55). The raw recorded videos were saved in a.TIF image stack at a resolution of 2048x2048 pixels (16 bit), resulting in a total video size of ~3.5 GB before processing.

### 2.5 Video pre-processing

To facilitate both faster and more consistent analysis, all phase contrast microscopy videos were pre-processed before being introduced to the machine learning pipeline. First, recorded videos were converted from 16-bit grayscale (2048x2048 resolution) to 8-bit grayscale and downsized to 1024x1024 *via* bilinear interpolation to reduce effective file size from ~3.5 GB to ~0.5 GB, allowing for faster image processing. The condensed videos were then histogram equalized to minimize brightness and contrast perturbations across video samples and throughout the duration of each video. The videos were then converted to 8-bit RGB frames and multiple image ‘sub-stacks’ were created *via* script such that each ‘sub-stack’ contained a number of frames that roughly matched the number of CPU cores present in the processing computer. Thus, for a 450 frame video being split among 18 cores, each generated sub-stack contained 18 images for a total of 25 sub-stacks. The ‘sub-stacks’ were subsequently saved in a folder structure that served as the primary directory for image input for the semantic segmentation process.

### 2.6 Machine learning models

**Figure 2:**
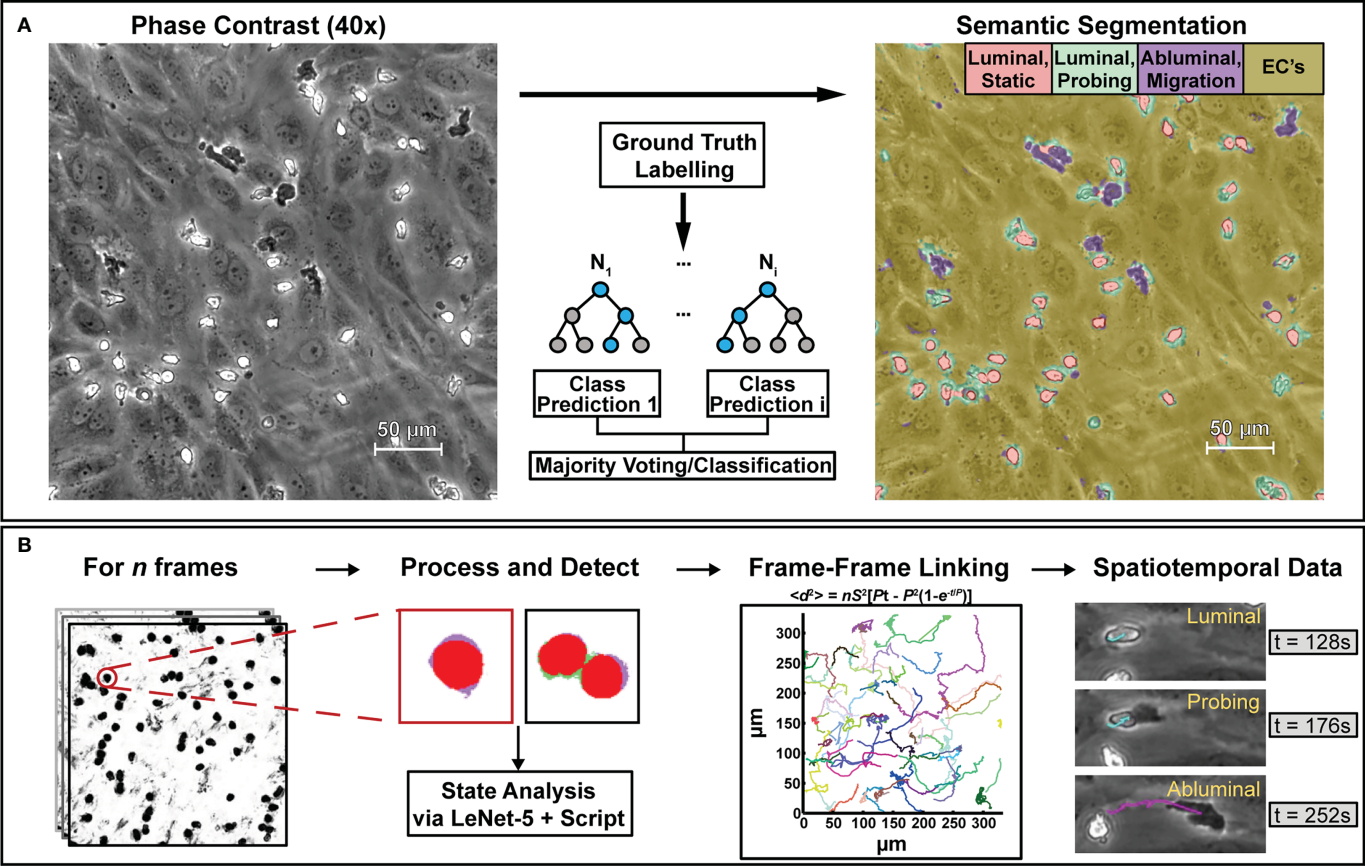
The computational methods described in this paper are designed to process phase contrast microscopy videos of human neutrophils interacting with vascular endothelial cell monolayers to extract bulk statistics such as neutrophil state and activity metrics. To achieve this, **(A)** select frames from phase contrast videos were taken and labelled with ground truth masks delineating neutrophil activity. Following labelling, a model based on a random forest algorithm was trained to provide pixel-level semantic segmentation of image datasets. **(B)** Segmentation maps were then taken and analyzed in script to separate all neutrophils from the endothelial background. Following this, another model based on the LeNet-5 CNN was trained to delineate single neutrophils from clustered ones. In conjunction with additional scripting, the CNN assists with counting, transmigration detection, and spatiotemporal analysis. Centroids obtained from PMN/cluster detection were then used to generate frame-frame particle trajectories *via* nearest-neighbor linking which, in conjunction with state information, allowed for spatiotemporal analysis of PMN behavior.

The overall workflow for the computer vision process can be seen in **Figure 2**. Two machine learning methods were utilized in this workflow, a semantic segmentation algorithm *via* random forest (**Figure 2A**) and classification algorithm *via* CNN (**Figure 2B**). More specifically, the implemented semantic segmentation algorithm uses a modified version of the random forest classifier called FastRandomForest (FRF) (36) while the CNN used is based off of LeNet-5 (37). Both were selected for speed and low hardware commitments, as the FRF model was trained in ~25 minutes while the LeNet-5 CNN was trained in ~15 minutes on an 8-core central processing unit (CPU), allowing for rapid iterations. Due to the speed of CPU training and the lack of available GPU libraries, GPU acceleration was not utilized for this study.

### 2.7 Model training for state analysis and validation

Following biological experimental data collection, additional negative and positive control datasets were recorded and used explicitly for training. Select images from both of the training negative and positive control data sets underwent ground truth labeling for the semantic segmentation approach *via* the “Trainable WEKA Segmentation” plugin (33) in FIJI (38), which utilizes the FRF algorithm. Images used for training were labeled with four cell classes *via* masking: apical/luminal “static” PMNs, apical/luminal “probing” PMNs, “basal/abluminal” transmigrated PMNs, and endothelial cells. Each class corresponded to a different discrete color in the masking layer, resulting in a 4-bin mask for the ground truth label. Beanshell scripts for the WEKA plugin were adapted from the ImageJ wiki and used alongside the ImageJ scripting language for automation of the training and classification processes. Data augmentation was incorporated into the model in order to increase PMN detection accuracy from variable recording conditions (e.g., blurs, brightness variations, etc.). The risk of overfitting with multiple features is noted to be low with random forest algorithms (39). The random forest model was retrained as needed until a satisfactory error rate was achieved (~2.68% *via* out-of-bag error calculated from training data).

After training, the semantic segmentation model was applied to n=3 (per condition, with or without fMLP) datasets that were not used in the training process and highest probability pixel maps were generated. The pixel maps were subsequently moved into another analysis script where all PMN-related detected pixels were extracted, morphologically linked *via* pixel association, and assigned a centroid. Segmented neutrophils were selected by searching for pixel clusters greater than 600 pixels in size, while all smaller detections were discarded as noise. Delineation between single neutrophils and clustered or multiple neutrophils is difficult *via* size or shape alone due to neutrophils presenting variable area and geometry depending on their state. To better count cells in a video on a frame-by-frame basis, a secondary CNN based on the LENet-5 architecture (40) was utilized. Briefly, select pixel segmentation frames from videos used for model training were taken and all PMN related features extracted and individually tabulated as either “single” or “multiple” neutrophils *via* ground truth labels. PMN detections from the segmentation maps were classified, and transformations (e.g., rotations, flips) were used to increase the number of training samples. In total, ~1,600 labeled samples were obtained for both classes for training. Training was performed with an Adam optimizer with 25 epochs and repeated with additional samples until a satisfactory error rate was achieved (~1.25% *via* confusion matrix on validation data). After separation into “single” and “multiple” categories, the precise number of PMNs in clusters were counted *via* additional scripting logic by dividing the pixel area of each cluster to the average pixel area of cells in the “single” PMN group on a frame-by-frame basis.

Transmigration ratios were determined by comparing pixel counts from the semantic segmentation map associated with the abluminal transmigrated PMNs to the luminal PMNs in a bulk manner post PMN detection and cleanup (i.e., the total number of pixels attributed to the transmigrated class were compared to the total number of pixels associated with luminal classes). Model validation was performed by checking for counting accuracy (<10% error with respect to manual counts) and transmigration ratio accuracy (within statistical significance of manual counts). Statistical significance for transmigration ratios were calculated by taking ‘equilibrium regimes’ (steady state) regions of PMN activity. Manual counting of PMNs was performed on the first frame of a video, followed by frame 30, then every 30th frame (i.e., 1, 30, 60, 90, …, 450) where both luminal and abluminal PMN populations were tabulated. For transmigrated PMNs, only fully transmigrated PMNs were counted for the purpose of comparison to the automated workflow results as PMN transmigration is a rapid event (~5 frames, or 20 seconds).

### 2.8 PMN tracking and validation

The PMN transmigration studies recorded for the state analysis were reanalyzed for tracking studies. Fifteen neutrophils were randomly selected and manually tracked from the three negative (-fMLP) and positive (+fMLP) control videos (n = 90 PMNs total) from the state analysis study and compared against a tracking script written in Mathematica. Frame-to-frame leukocyte trajectories were created using the nearest neighbor method (41) of particle linking by detecting PMN centroids from semantic segmentation data, where the closest detections from one frame to another are associated and subsequently linked as a trajectory. Centroid detection was performed with feature extraction algorithms built into Wolfram Mathematica that are also commonly available across multiple platforms [e.g., image moments and invariants (42, 43)]. Additional scripts were written to mitigate tracking issues in incidents when multiple PMNs overlap or cluster in a video frame, correct errors attributed to the nearest-neighbor linking algorithm *via* proximity thresholding, and assess population subgroups such as transmigrated PMNs only and non-moving PMNs. For clustered PMNs that localized in proximity either luminally or abluminally, all trajectories associated with the PMN “bulk body” were assigned a common centroid while PMNs remained in close association with each other. A size threshold was incorporated to ensure only large clusters of PMNs follow this linking logic. Upon PMN overlap (luminal crawling over abluminal), tracks were linked to a common centroid and subsequently restarted upon the end of the PMN overlap event, although individual PMN identity is not always preserved, and this resulted in the generation of track fragments. To prevent tracks from jumping across the video, the nearest-neighbor linking approach incorporated mechanisms to stop linking by both preventing the same centroid from being used in multiple tracks (unless clustering occurred) and by limiting the search for new trajectories by a threshold of 55 pixels radially over 5 frames (11 pixels added per future frame search). PMN motility was characterized by calculating meandering index, defined as displacement divided by total path length (44). Final data generated included leukocyte spatial location, persistence, and mean squared displacement based on fitting the Dunn equation (45) with a time gap of 120 seconds using track fragments >100 frames in length. The 15 longest tracks for each condition, as well as a random group of 15 tracks with track length greater than 100 frames were also used in curve fit calculations. Tracking validation was performed by comparing both speed and persistence values for statistical agreement with manual tracks *via* a Students t-test.

Population subgroup analysis was facilitated by combining state information from the semantic segmentation workflow and tracking data. Trajectory fragments were combined with state information to create a data structure that saw track fragments reorganized into two groups. One representing tracks associated with luminal PMNs/PMN clusters, and another associated with abluminal PMNs/PMN clusters. A pixel ratio of greater than 50% transmigrated class detections was used as the threshold for counting a PMN/PMN cluster as an abluminal detection. Given the dynamic state of leukocytes in these microfluidic devices, additional track ‘sub fragments’ were generated to split trajectories if PMNs transmigrated (luminal to abluminal). Curve fitting and parameter extraction followed for the two groups of tracks in a similar fashion to the rest of the tracking analysis. A validation of this methodology (via comparison to manual labelling) was performed by assessing the bulk state of a trajectory fragment. The average error in state detection for a full track fragment was 5.72%.

### 2.9 Statistical analysis

All cell culture experiments were performed with twelve replicates (each in individual µSiM devices) for a total of n=6 for both negative and positive control studies. Experiments were performed on different days, using sequential passages of HUVECs. Three datasets from both conditions were used in ground truth labelling (by selecting a fraction of frames) while another n=3 studies from both experimental groups were used for model analysis and validation. For model validation, bulk distributions were evaluated with a two sample Kolmogorov-Smirnov (KS) test, individual replicates were assessed *via* Student’s t-test, and multiple comparisons were done with a One-Way ANOVA. All graphical results are reported as mean ± standard error of mean (SEM). Statistical analysis was performed in Prism (Graphpad Software Inc, San Diego, CA).

### 2.10 Computational resources and scripting

All image processing and model deployment was performed on an 18-Core/36-thread (Xeon W, Intel Corporation, Santa Clara, CA) iMac Pro (Apple Inc., Cupertino, CA) with 64GB of RAM. Scripting, coprocessing, and model training was performed on an 8-core/8-thread Mac Mini (M1, Apple Inc., Cupertino, CA) with 8GB of RAM. A key characteristic of this workflow is its lightweight nature, as none of the ML processes utilized in this study incorporated graphics processing units (GPUs) and were capable of rapidly running on central processing units (CPUs) alone. For the semantic segmentation approach, a random forest algorithm was used for analyzing frames from experimental videos *via* the WEKA module incorporated into FIJI known as ‘Trainable WEKA Segmentation’ (33). The WEKA module was controlled *via* Beanshell (46) and ImageJ macro language scripting for both training and classification. Ground truth labels were created with a GUI interface built into FIJI. For *post-hoc* analysis of semantic segmentation data *via* convolutional neural network and common image analysis algorithms, additional scripts were written in Mathematica (Wolfram Research, Champaign, IL). Specifically, Mathematica was utilized to initialize, train, and deploy a custom LeNet-5 (40) alongside common feature extraction algorithms to facilitate the analysis of semantic segmentation data. For the LeNet-5 model training, a typical 80/20 training/validation split was utilized. Further, Mathematica was utilized for the creation of a nearest-neighbor linking approach for particle tracking. Data management and workflow was managed with bash scripting.

## 3 Results

### 3.1 Machine learning model training results

After training the machine learning models, the error for the WEKA based FRF algorithm and secondary LENet-5 algorithm were ~2.68% and ~1.25% respectively. Error for FRF is calculated using the out-of-bag error rate while the secondary LENet-5 based model calculated error from its associated confusion matrix. This accuracy facilitates the secondary analysis, where the FRF algorithm provided high contrast imaging information (**Figure 2A**, additional examples in S1) and the LeNet-5 algorithm applied to segmentation data was used to facilitate population counting, transmigration ratios, and tracking analysis (**Figure 2B**). Finalized reports are generated that contain spatio-temporal state information associated with a PMN track.

### 3.2 Population assessment

**Figure 3:**
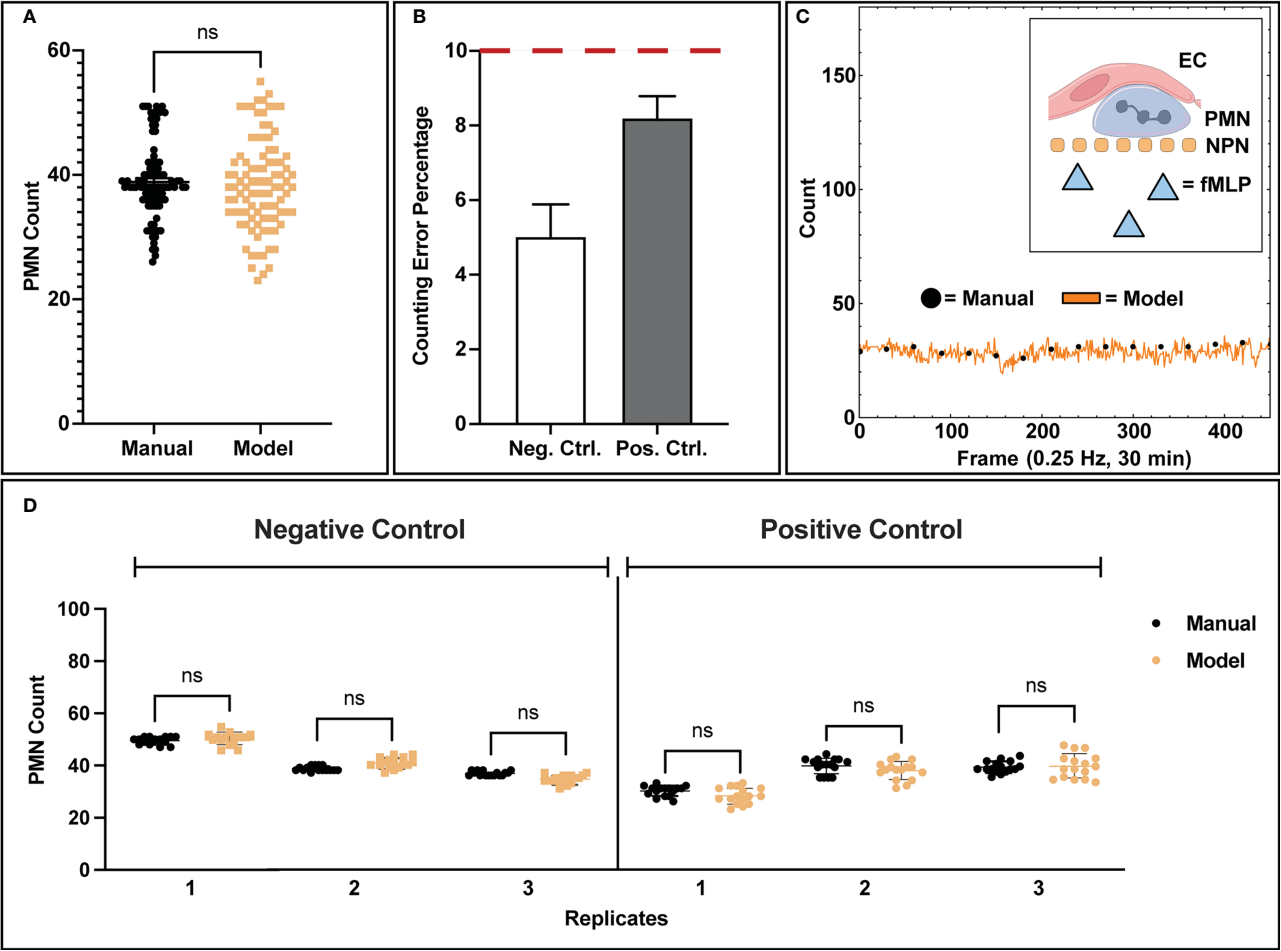
Analysis of model counting capability. **(A)** Manually tabulated counts were compared against the model process *via* statistical testing (Two-Sample KS Test) and were found to have similar underlying distributions. **(B)** When comparing control conditions individually, negative control studies presented lower error rates in counting (5.02%) versus positive control studies (8.20%), which is likely due to the increase in amorphous morphology seen in activated PMNs. **(C)** Example data showing temporal population detection (both manual and model based) for a positive control study. For the 30-minute duration of each control study, no PMN population loss or gain was observed from the recording field of view, indicating that PMN population distribution is homogenous. PMNs exposed to fMLP concentration gradients are unable to cross the NPN membrane. **(D)** Comparisons of model counting capability for individual replicates. Each data point represents a successive frame in a video/experimental condition where population counts were tabulated. In each replicate for both positive and negative control studies, the model shows statistically similar counts to manual counting as well as a tight clustering for all the associated data points. Statistics: ns, not significant.

After segmentation, a counting script provided an estimate of the number of PMNs present in a video frame alongside centroid markers for future tracking detection. When comparing the population detections in frame from the model versus manual counting, the bulk distributions of all analyzed frames were found to be statistically similar (**Figure 3A**) with comparable clustering, indicating that the model predictions are in line with manual observations. Note, a negative and positive control were used for all comparisons made in this study. For positive control studies, 10 nM fMLP was introduced into the bottom channel of the µSiM device to create a chemokine gradient. When assessing each control condition individually, we find that the model is capable of counting PMNs with less than 10% error (**Figure 3B**) compared to manually tabulated results. Notably, neutrophils in negative control studies are more reliably counted with ~5% error versus positive control studies at ~8% error. This can be attributed to the increased difficulty in detecting activated neutrophils as they typically present amorphous or irregular morphologies in comparison to the rounded shapes presented by non-activated neutrophils.

With our typical magnification (40x objective), only ~7.8% of the available membrane surface area is imaged. This limited field of view introduces the possibility of bias if PMNs leave a recording boundary. A key characteristic of these experiments, however, is that PMN populations on the membrane surface were found to be stable for the 30-minute experimental duration (**Figure 3C**), eliminating potential bias *via* population loss from the limited recording field of view. In negative control studies, this can be explained by the lack of stimulus and thus relatively inactive PMNs. For positive control studies, the presence of a chemokine in the bottom chamber activates neutrophils but the nanoporous membrane prevents them from migrating into the bottom channel containing the chemokine source. Thus the result is again a steady number of migrating PMNs over time in the 30-minute experiments. To complement this bulk analysis, individual experimental replicates were analyzed for population counting accuracy (**Figure 3D**). For each experimental replicate analyzed by the machine learning workflow, the automated process depicted both high accuracy in counting and tight population clustering, which is indicative of population stability over time. Variability in population seeding density between experiments was seen and likely occurs due to inaccuracies in the small volumes (~20 µL) injection of PMN-containing media into fluidic channels.

### 3.3 Transmigration analysis

**Figure 4:**
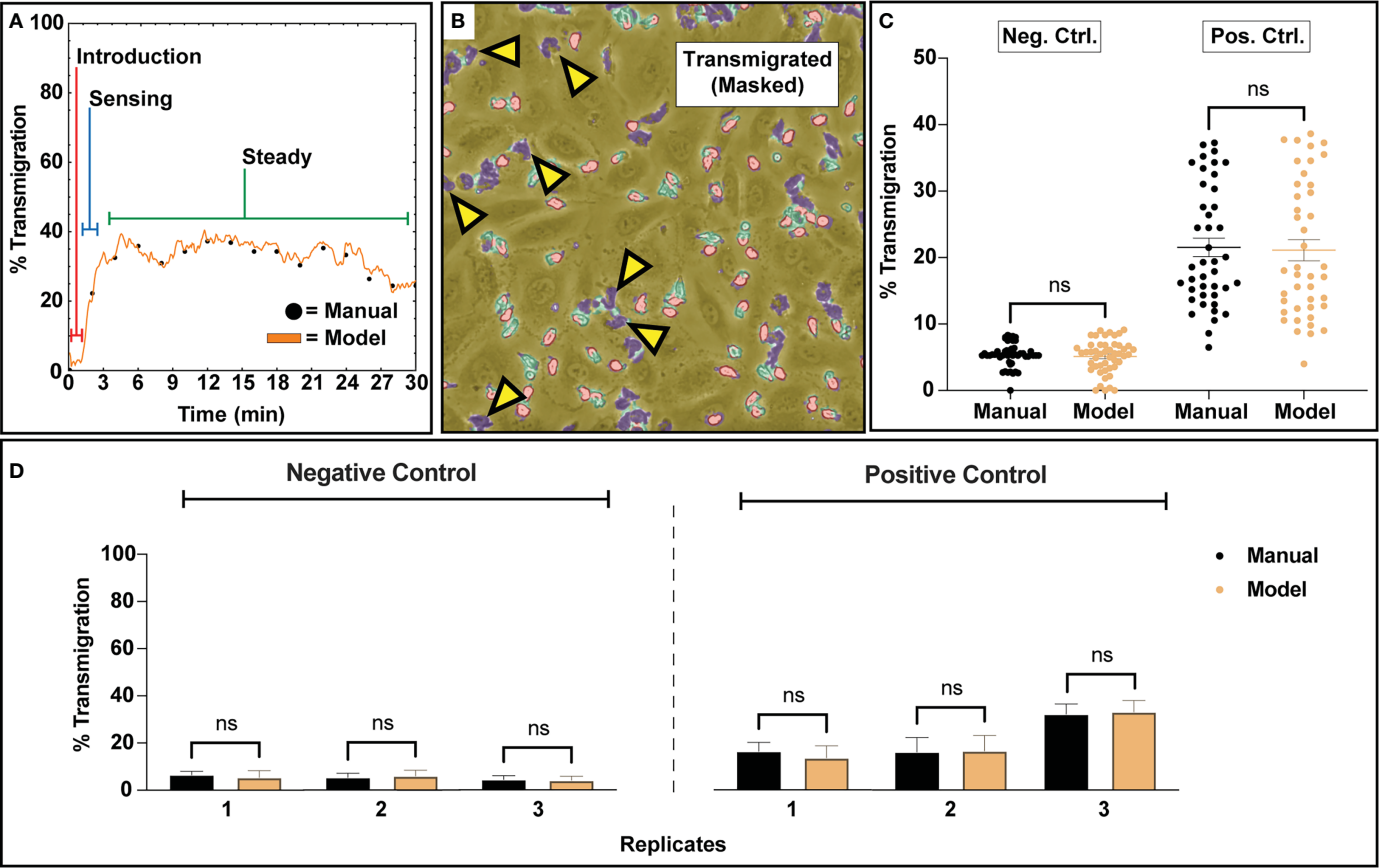
Transmigration analysis for assessing subgroups of PMNs that respond to a 10 nm fMLP chemical stimulus along a concentration gradient. **(A)** A typical example of PMN transmigratory behavior in a positive control study. Upon introduction to a device, PMNs appear inert and slowly start to acclimate to their new environment. Within a few minutes, escalating transmigration begins to occur, ultimately reaching a ‘steady state’ where a portion of the PMN population maintains transmigratory behavior. For the purposes of comparing transmigration ratios, an average of the ‘steady state’ regime was utilized. **(B)** An example figure from a positive control training set study depicting a ‘steady state’ segmentation map overlayed onto a corresponding video frame. Despite the usage of fMLP, a potent neutrophil chemotactic agent, only a portion of the PMN population responds to stimulus. **(C)** An analysis script calculated transmigration ratios for analyzed video frames from n=3 negative and positive control studies. The final results were compared to manually tabulated results, where a PMN was considered transmigrated only if it was fully phase dark. For both experimental groups, when comparing all analyzed frames, bulk distributions were found to be statistically similar *via* Two-Sample KS Testing. As expected, positive control studies experienced higher transmigration ratios due to the usage of fMLP as a chemoattractant. **(D)** Comparison of the average transmigration ratio from ‘steady state’ regimes from temporal data for individual replicates versus model counting. For each replicate, statistical similarity is achieved between manual and model based counting. Statistics: ns, not significant.

The segmentation map generation procedure combined with the counting algorithm facilitated transmigration analysis by providing context on PMN state. Negative control studies (no stimulation) displayed minimal PMN transmigration activity, where <5% of PMNs were actively engaged in transmigratory behavior. In contrast, positive control studies stimulated by a transmembrane gradient of fMLP displayed robust PMN transmigration ranging from ~10-40% average ratios between three independent experiments. Across all positive control studies, bulk PMN transmigration displayed a typical behavior consisting of no activity upon PMN introduction into the device, a sensing regime where PMNs begin to rapidly transmigrate, and then a pseudo ‘steady’ regime where transmigration behavior is maintained for a subset of the PMN population (**Figure 4A**). For the purposes of analyzing this behavior, all comparisons made for the positive control groups only account for the subset of transmigration data generated during the steady state regime. A typical example of multiple transmigrated PMNs (with highly amorphous morphologies) co-existing with non-activated PMNs (with rounded morphologies) on an endothelial surface is shown in **Figure 4B**. For both conditions, the bulk distributions for all analyzed frames are found to be statistically similar for both model and manual counting (**Figure 4C**). As expected, positive control studies solicited higher transmigration ratios than negative control studies due to the potent nature of fMLP as a PMN chemoattractant (**Figure 4C**). Notably, the high variability of transmigration ratios in fMLP gradient devices, along with an apparent ceiling of ~40% transmigration, indicates a heterogenous response to this chemotactic factor within the PMN population. This becomes more evident when viewing results from individual replicates, where manual counting agrees with both the mean model prediction and the variation about the mean (**Figure 4D**). While negative control studies typically presented low transmigration ratios, positive control studies display experiment-to-experiment variability.

### 3.4 Counting and transmigration error analysis

**Figure 5:**
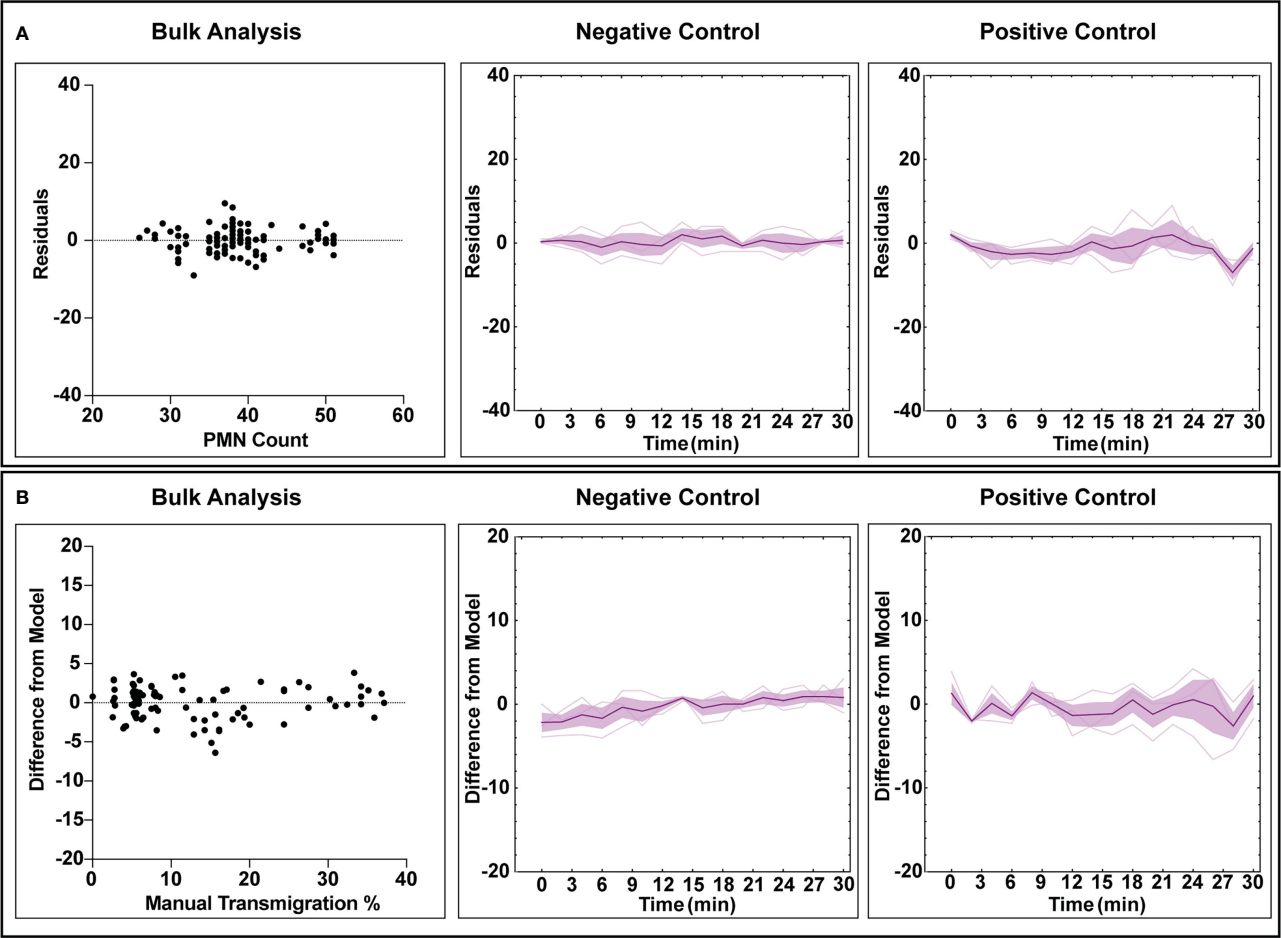
Error analysis for counting and transmigration detection for both bulk measurements (checking each analyzed frame) and temporally over the course of multiple experiments. For the time associated graphs, n = 3 independent experiments utilizing individual devices are depicted. The dark purple lines represent time-dependent mean, shaded regions represent standard error of mean, and light lines represent individual replicates. **(A)** Residuals generated from the counting analysis are evenly dispersed, indicating that the automated workflow has no bias towards overcounting or undercounting. Similarly, this behavior is maintained when looking at all experimental replicated in a time-dependent manner, with positive control studies displaying more variability over time. **(B)** Bulk analysis for transmigration displays a lack of undercounting or overcounting bias when compared to manually tabulated data. Time-dependent data continues to display this trend, with positive control studies again displaying higher variability with experiment time. For both counting and transmigration, positive control studies see higher error with time due to increased PMN amorphous morphology and clustering events.

The automated workflow demonstrates statistical parity to manual counting with low error rates for both negative and positive control studies. To better understand error rates, analyses were performed to assess under/over counting as well as time-dependent error caused by increasing PMN morphological changes and clustering. For population counting, comparisons were made by plotting residual plots in bulk and time associated data for both experimental conditions (**Figure 5A**). In this context, a residual plot displays the difference between computer and manual measurements. Transmigration data was assessed similarly, with ‘difference from model’ used instead of discrete counts (**Figure 5B**). Note, ‘difference from model’ refers to the difference of percent transmigration values and is effectively comparing residuals. Also, all transmigration data was considered for this study instead of using the ‘steady state’ only (**Figure 5A**), as we are evaluating overall model performance. For bulk counting and transmigration analysis, the residuals are well dispersed and do not indicate any bias towards under or over counting when comparing the automated process to manual counting. Temporal data for both counting and transmigration illustrates a similar conclusion, with less error in both categories for negative control studies versus positive control ones. As time increases, error increases in positive control studies which can be attributed to the increased difficulty in detecting PMNs with irregular shapes. Regardless, the automated workflow exhibits high accuracy counting and transmigration detection of PMNs in µSiM devices.

### 3.5 Tracking analysis

**Figure 6:**
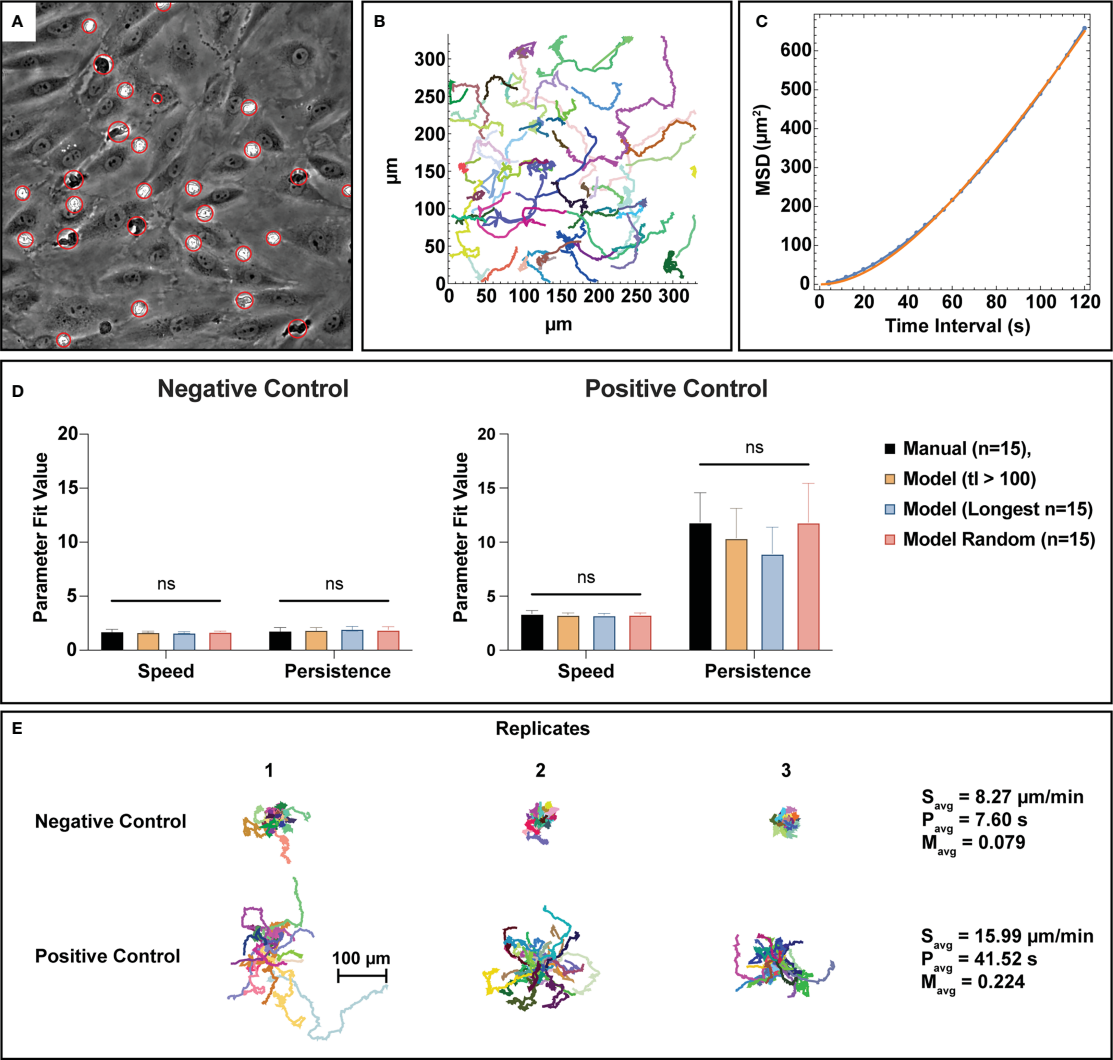
Bulk tracking statistics can be extracted from semantic segmentation data utilizing a nearest neighbor linking tracking methodology. **(A)** PMN and PMN cluster centroids are gathered from the automated cell counting process and organized into trajectory fragments, the subfigure depicted here displays a visual representation of the generated centroids. Each red circle has a different size that corresponds to the equivalent disk radius of the detected PMN or cluster. **(B)** A graphical example of track fragments plotted in an xy plane where each migration path is assigned a random color. Tracking logic and constraints are detailed in the methods section. Combined with segmentation data, the track fragments can provide spatiotemporal information on PMN behavior. **(C)** To better understand bulk PMN activity behavior in the µSiM device, track fragments were fit to the Dunn equation using a time interval τ=120 seconds, resulting in the extraction of both average speed and persistence. This subfigure shows a typical example of a Dunn equation fit, where the blue dots represent individual data points and the orange line represents a curve fit. **(D)** Speed and persistence were compared between n=15 manual tracks, track fragments with a length of 100 frames (400 seconds) or more, the n=15 longest tracks, and n=15 random track fragment for both negative and positive control groups. For all parameters, statistical parity was achieved with noticeably higher speeds and persistence values for the positive control group. Persistence measurements in the positive control study appear to be inversely correlated to track length, however the use of random tracks seems to resolve this issue. **(E)** Spider plots using a universal origin depict the contrast between low activity, unstimulated PMNs and high activity, stimulated ones. In general, PMNs exposed to fMLP are faster, more persistent, and have higher meandering indices versus unstimulated ones. Statistics: ns, not significant.

One of the outputs from the automated counting process is centroids for all detected cells and clusters. This data was used to create neutrophil tracks *via* a nearest neighbor linking based tracking algorithm. For this study, only bulk characteristics (average speed, persistence, and meandering index) were assessed to obtain useful information on the activity levels of the total PMN population. Speed is a measure of displacement over time, persistence is a measure of time spent before changing direction, and meandering index represents the linearity of a trajectory. An example of a centroid overlay map from the cell counting process can be seen in **Figure 6A**. After centroid collection, tracks were linked based on criteria described in the methods section and can be displayed in an x-y coordinate map, where each color corresponds to a different track fragment (**Figure 6B**). To evaluate tracking performance, the generated track fragments were then processed to calculate the mean-square displacement (MSD) over time and fit to the Dunn equation to extract speed and persistence assuming the trajectories are a semi-persistent random walk (47) (**Figure 6C**). Analyzing the MSD over 120 seconds in each case, this method produced a mean speed and persistence for each trajectory. For negative control studies, both speed and persistence were found to be statistically similar between manual tracking and all assessed track fragment groups created from the automated process (**Figure 6D**). Similarly, positive control groups display statistical similarity between manual and automated parameter measurements, however there is noticeably more variability in the persistence measurements. Automated persistence measurements appear to be inversely correlated to track fragment length where longer tracks result in lower persistence values, indicating a potential form of mathematical bias. It is important to note however, that there is no statistical difference between persistence measurements for the assessed track fragment groups, and the usage of random tracks in analysis appears to resolve this phenomenon. All tracks from the automated process can be organized into spider plots, where a universal origin is set (**Figure 6E**). When comparing tracks in this manner, unstimulated PMNs demonstrably crawl less on an endothelial surface versus those exposed to an fMLP gradient in the positive control studies, which is in line with physiological expectations. Unstimulated PMNs are slower overall, less persistent, and have lower meandering indices than their stimulated counterparts (**Figure 6E**). Note, meandering index measurements measure path linearity on a scale from 0 to 1 and are similar between manual and machine measurements (S2).

### 3.6 Subgroup analysis and summary

**Figure 7:**
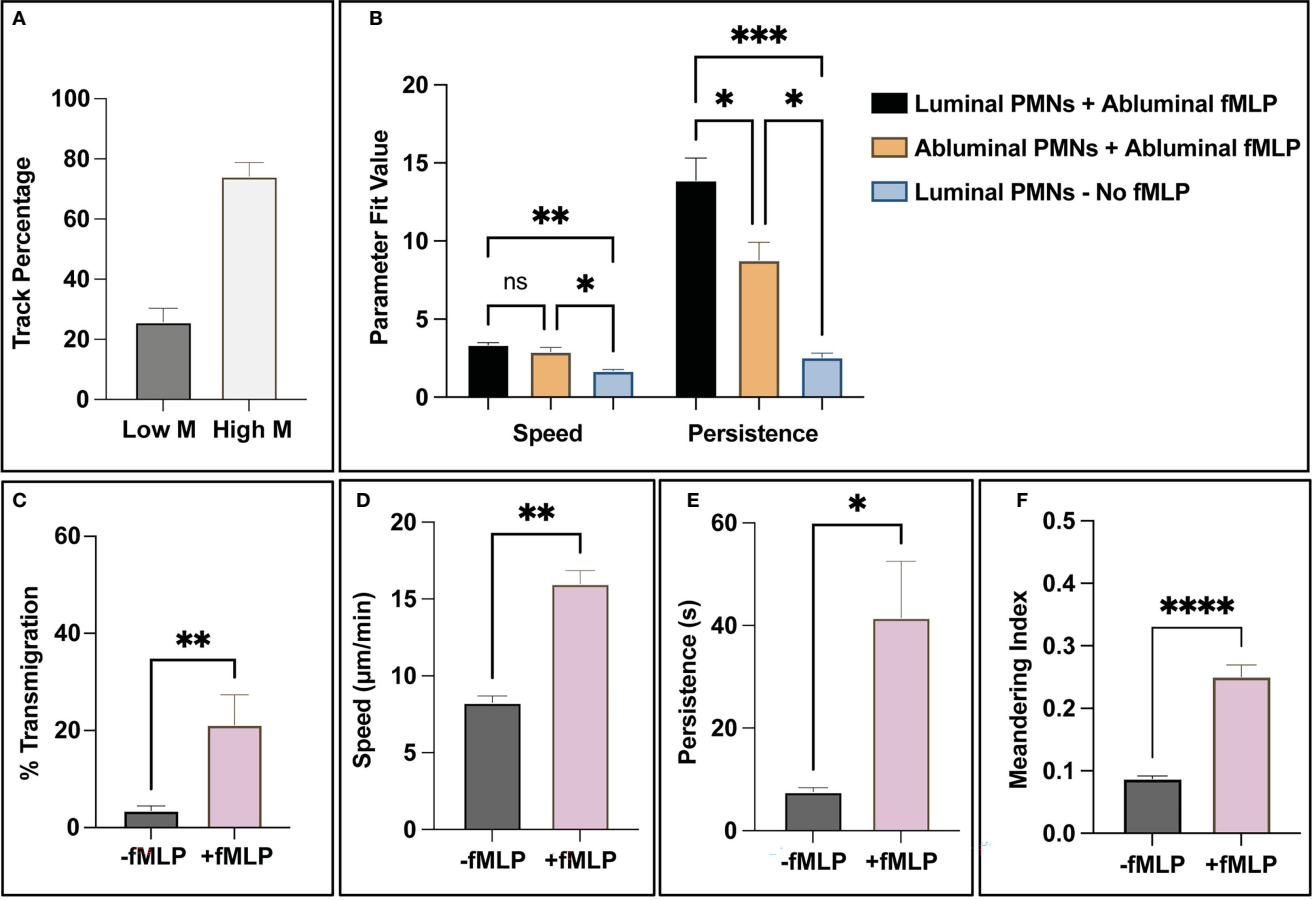
The combination of PMN state detection and tracking ability allows for the creation of spatiotemporal data and subsequent analysis of PMN population subgroups. **(A)** For positive control studies, all trajectory fragments were analyzed for relative movement *via* meandering index. Notably, a small subgroup of ~30% analyzed track fragments had limited (<0.1 meandering index) movement in response to fMLP, compared to the 70% that showed a more robust response. **(B)** When addressing PMN statistics for luminal vs abluminal groups, the presence of a chemotactic agent appears to dictate overall speed. (Note: too few PMNs transmigrated in negative control studies, < 5% on average, for meaningful conclusions to be made). Transmigrated PMNs in positive control devices (+fMLP) are less persistent than PMNs localized on the luminal surface of the endothelium. **(C-F)** As a final summary, the automated workflow detects statistically significant differences between unstimulated and stimulated PMNs. In the studies that incorporated fMLP, PMNs transmigrated more frequently, were faster, more persistent, and had higher meandering indices. All of these observations are expected and correlate with literature. Statistics: *P < 0.05, **P < 0.005, ***P < 0.0005, ****P < 0.0001.

With validated state analysis and tracking capabilities, the computational tools described in this study are capable of generating spatiotemporal data profiles on all PMN trajectories. One behavior evident from observing PMNs in positive control studies is that a subset of the population does not respond to fMLP stimulus. Using the meandering index measurement (displacement over path length) and setting a threshold of <0.1 as a “non-reactive” trajectory, ~25% of trajectories mapped displayed little movement (similar to negative control studies) in contrast to ~75% that displayed robust movement across all positive control studies (**Figure 7A**). When assessing the effect of directional localization on PMN speed and persistence, a few notable behaviors were observed. First, fMLP stimulated PMNs have similar speeds regardless of location with respect to the endothelium and are significantly faster than non-stimulated PMNs (**Figure 7B**). The location and stimulation status of a PMN greatly effects persistence, noting that stimulated and luminally positioned PMNs are significantly more persistent than stimulated and abluminally positioned ones (**Figure 7B**). Both stimulated groups of PMNs are significantly more persistent than unstimulated PMNs (**Figure 7B**). Further study of PMN subgroups may help elucidate mechanisms attributed to PMN heterogeneity. Lastly, as a summary, fMLP stimulated PMNs transmigrated more, were faster, more persistent, and had higher meandering indices (**Figures 7C–F**). All of these results are expected and correlate with literature on PMNs across both human and animal models (48–51).

## 4 Discussion

MPS are increasingly being used to model vascular systems (22, 23), and they have facilitated the exploration of mechanisms of human disease because of their ability to replicate vascular physiology with human cells. The attention placed on essential physiology, in conjunction with the ability to rapidly evaluate acute vascular barrier dysfunction in a high-throughput format, enables MPS to serve as platforms for discovering future pharmaceutical interventions (25) with high physiological relevance. In our lab, we have created the ‘µSiM’ platform (27) that complements endpoint studies such as ELISA or immunofluorescence with the ability to rapidly collect high-resolution temporal imaging data at the endothelial interface. This is due to the incorporation of optically transparent, highly porous nanomembrane materials that allow for the monitoring of PMNs on the endothelial interface without the incorporation of exogenous dyes. Manually analyzing high-content imaging data is difficult however because of the size of the imaging datasets (31) and may fail to capture the dynamism of PMNs transmigrating through vascular endothelium because of user bias. Here we demonstrate that an automated process using machine learning in conjunction with computer vision techniques is capable of obtaining bulk metrics of PMN activity in response to a chemokine gradient stimulus.

In this study, we used our µSiM microvascular mimetic to image different PMN responses to chemical stimulus *via* incorporation of fMLP gradients originating in the abluminal or ‘tissue compartment’ of our device. Through the adaptation of two machine learning algorithms, a semantic segmentation approach *via* random forest and classification *via* CNN, we were able to extract bulk metrics and data from the devices. Specifically, we were able to accurately ascertain the number of PMNs present in a field of view, assess bulk transmigration activity, and gather bulk activity metrics based on a nearest-neighbor-linking tracking algorithm. Our automated results are statistically indistinguishable from results obtained with manual counting. The automated approach is computationally efficient, accurate, and modular. Training the models used in this study takes ~40 minutes on an 8-core processor, and deployment to achieve a full report on a single experiment is accomplished in ~2.5 hours on an 18-core processor. Because of the CPU-centric nature of our analytical process, this workflow is accessible for groups without extensive computational resources. Importantly, GPUs are not necessary but are available as a resource for accelerated computing *via* CLIJx for the WEKA based semantic segmentation and cuDNN for the CNN based classification scheme. In the future, such acceleration may be required to process higher frame-rate videos.

There are some limitations on the level of detailed information that can be obtained using the automated workflow we have developed. Notably, our ability to detect intermediate PMN transmigration phases is limited because of the rapid (~5 frames or 20 seconds) nature of a transmigration event. While this may be addressed with higher frame rate capture, additional computational resources would be required to handle the increased amount of imaging data. Secondly, the tracking methodology described in this study is conservative and generates multiple track fragments instead of a full trajectory that maintains PMN identity. While bulk measurements of PMN speed, persistence, and meandering index are not affected by this, the lack of tracking cell identity limits the ability to detect events such as PMN reverse transmigration, which is increasingly implicated in the pathophysiology of diseases such as sepsis (52). While bulk measurements of the PMN transmigration ratio can provide context for this event occurring inside of a device (e.g., ratio trending downward after a local maximum), the loss of additional tracking/identity information may also limit the ability to observe additional phenomena such as hot spots for transmigration. The incorporation of probabilistic techniques and global optimization as recently described in Vladymyrov et al. (53) can be utilized to overcome this limitation, but with additional computational costs.

Despite these limitations, the computational tools established in this study pave the way for automated or semi-automated workflows that quantify immune cell dynamics in microvascular mimetics. This tool could eventually be used with patient-specific cells in clinical diagnostic assays or for following a patient’s response to treatment (54). The workflow could also become part of development pipelines for pharmaceutical products. One of our research interests is characterizing the asymmetric EC response to directional inflammatory stimulus and its consequences for immune cell trafficking. Understanding the role of apicobasal polarity in pathogenesis is imperative as there is an increasing body of evidence that vascular barrier dysfunction and subsequent immune cell transmigration is implicated in diseases such as sepsis (1), Alzheimer’s disease (2), and MS (3). As an example, MS disrupts BBB tight junctions, resulting in the relocation of abluminal CXCL12 towards the luminal vasculature (55). This loss in apicobasal polarity leads to increased leukocyte recruitment, potentially contributing to neuro-injury as increased recruitment of leukocytes to the CNS is associated with a host of negative cognitive effects (56–58).

Prior studies in our lab have also demonstrated different EC responses when stimulated *via* luminal or abluminal exposure to the cytokine TNF-α, modeling systemic vs. localized sources of inflammation respectively (24). We found a that luminal treatment of ECs in a µSiM vascular mimetic resulted in a biased secretion of the chemokine IL-8 towards the luminal or ‘blood side’ of the device, while abluminal TNF-α exposure produced an approximately uniform secretion into the blood and ‘tissue sides’ of the device. Both forms of stimulation resulted in luminally oriented surface expression of the leukocyte adhesion molecule ICAM-1. Interestingly, the abluminal TNF-α exposure resulted in significantly higher PMN transmigration rates compared to negative controls and luminal exposure. This suggests that inflammatory signals arising from inflamed perivascular tissue result in a stronger recruitment of immune cells than those arriving through the circulation. These insights followed from many hours of labor-intensive manual tracking of PMNs in 30-minute time-lapse movies. Thus our motivation for the ML tools we present here stems from first-hand experience that the rate of biological discovery can be significantly accelerated by the use of automated methods for the analysis of high content imaging data.

Another important application of the tools presented here is in the analysis of functional heterogeneity in leukocyte populations. Originally thought to be of a single type, there is an increasing appreciation that PMNs are heterogenous in function and outcome (59–61). This observation coincides with a growing number of studies indicating that PMN dynamics such as polarization or reverse transmigration are implicated in multiple disease pathologies, including sepsis (13, 18, 62) and cancer (63, 64). While the delineation of such PMN “subgroups” typically involves genomic analysis (e.g., single cell RNA-seq) or flow cytometry (65), we observed the presence of possible population subgroups through trajectory analysis alone. Meandering index measurements identified small populations of PMNs that failed to respond to fMLP stimulus (**Figure 7A**) and differences in motility parameters were observed for PMNs depending on their localization with respect to the endothelium. These examples may indicate intrinsic population heterogeneity and transmigration-induced behavioral differences, respectively. We observed that luminally localized PMNs stimulated with fMLP appeared to be more persistent than their abluminal counterparts (**Figure 7B**). In agreement with prior data analysis performed manually (24), PMN speeds were found to be similar between luminally and abluminally localized fMLP-stimulated PMNs, indicating that the differences in migratory behavior relate to direction sensing rather than cell movement. In the future, more experiments can be performed to investigate PMN heterogeneity particularly in the presence of inflammatory cytokines that simulate diseased endothelium, and/or with the incorporation of other cell types (e.g., pericytes) in co-cultures to better mimic *in vivo* physiology.

## Data availability statement

The raw data supporting the conclusions of this article will be made available by the authors, without undue reservation.

## Ethics statement

The studies involving human participants were reviewed and approved by Research Subjects Review Board, University of Rochester. The patients/participants provided their written informed consent to participate in this study.

## Author contributions

SA performed biological studies, code writing, and manuscript writing/editing. JM, RW, and MC assisted with experimental direction as well as both manuscript writing and editing. All authors contributed to the article and approved the submitted version.
