# Supplementary material for: A computer vision approach for analyzing label free leukocyte trafficking dynamics on a microvascular mimetic
Source: Front Immunol. 2023 Mar 24;14:1140395. doi: 10.3389/fimmu.2023.1140395 (PMC10080102; doi:10.3389/fimmu.2023.1140395)
Supplement: Supplementary file 1 [file DataSheet_1.docx]

Supplementary Material

A Computer Vision Approach for Analyzing Label Free Leukocyte Trafficking Dynamics on a Microvascular Mimetic

S. Danial Ahmad^1^, Mujdat Cetin^2,3^, Richard E. Waugh^1^, James L. McGrath^1*^

^1^University of Rochester, Department of Biomedical Engineering, Rochester, NY, USA

^2^University of Rochester, Department of Electrical and Computer Engineering, Rochester, NY, USA

^3^University of Rochester, Goergen Institute for Data Science, Rochester, NY, USA

*** Correspondence:**James L. McGrath
jmcgrath@bme.rochester.edu

# Supplementary Figures

#
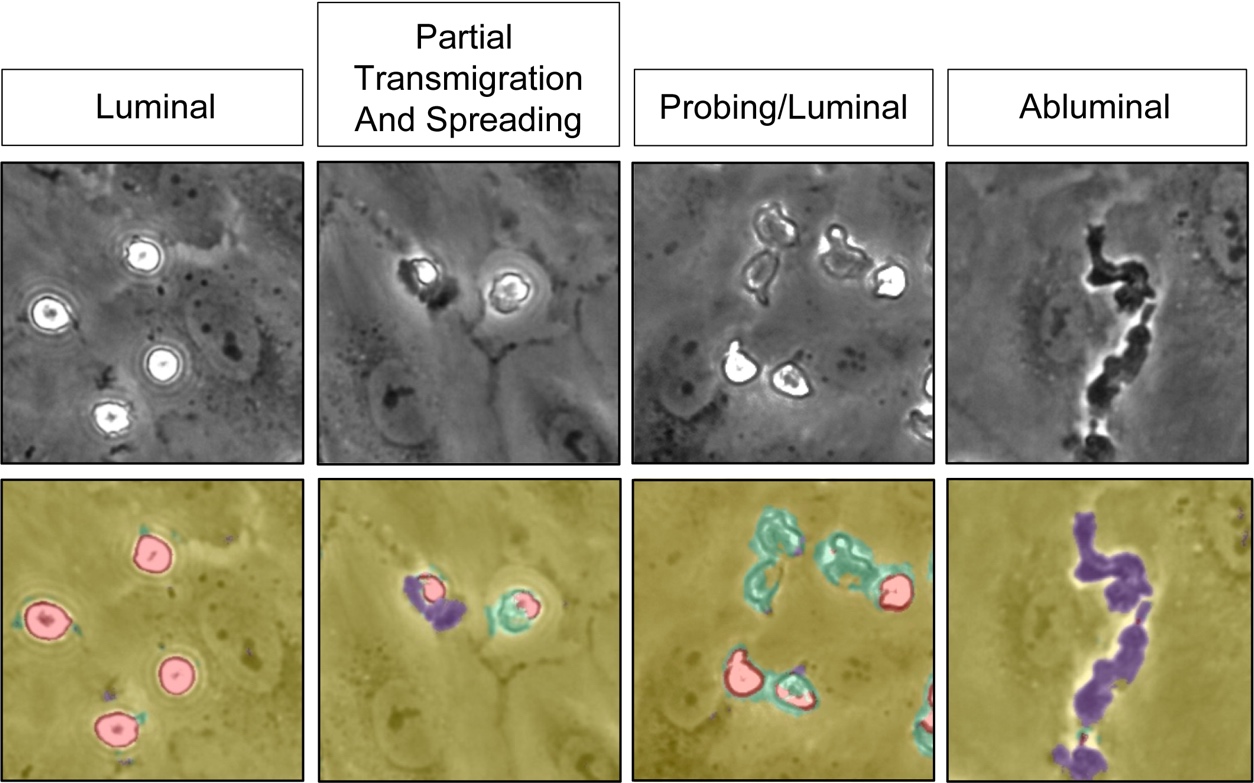


# Supplementary Figure 1. Additional examples of labelled PMNs following the color scheme noted in Figure 2A. The semantic segmentation algorithm distinguishes pixels associated with luminal (red), probing/spreading (green), and abluminal (purple) sections of PMNs as they navigate the HUVEC endothelium.


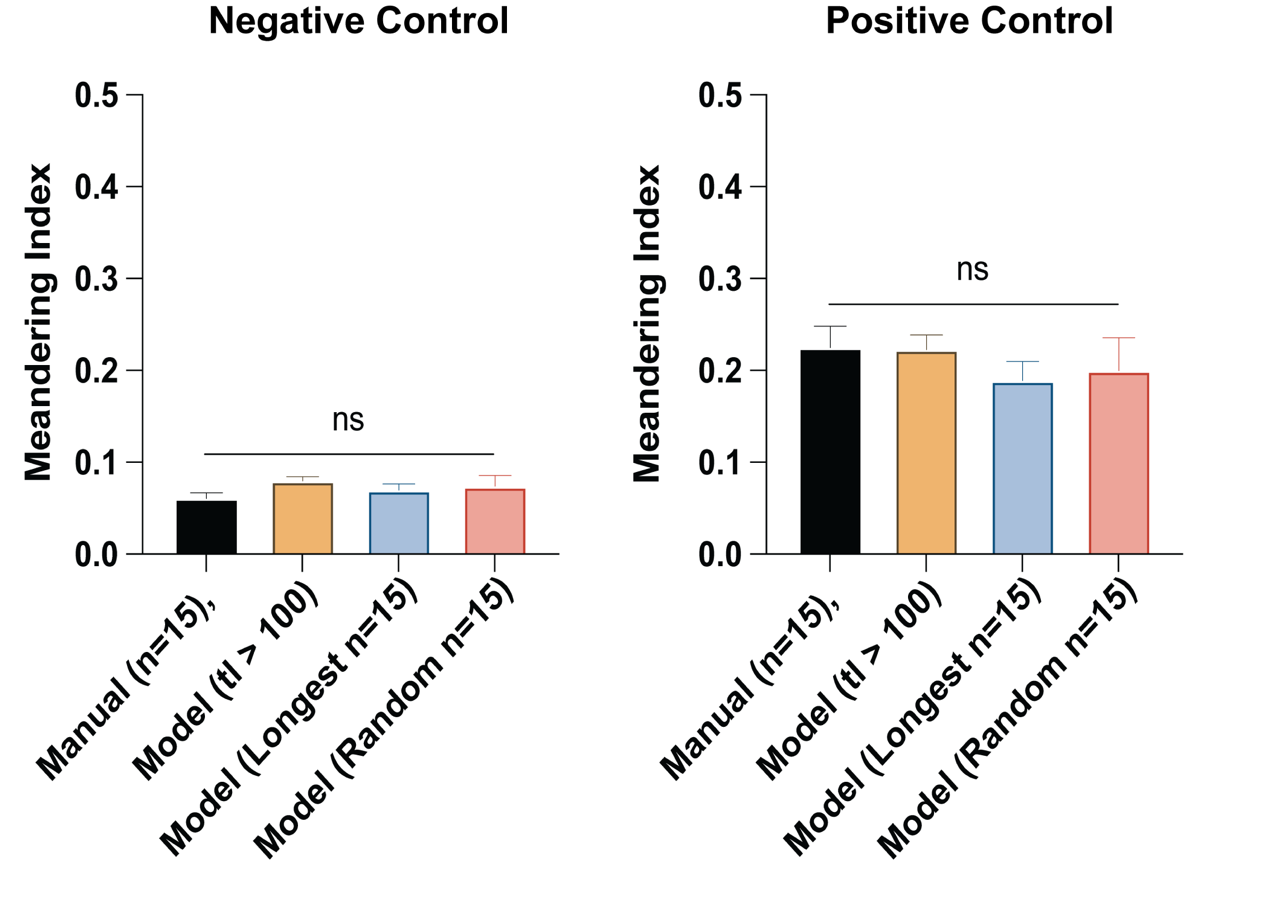


**Supplementary Figure 2.** Verification of meandering index results, which assess path linearity on a scale from 0 to 1. Meandering indices were compiled for all manual tracks and machine generated track groups for both positive and negative control studies. For both experimental conditions, meandering indices were found to be statistically similar when comparing model tracking to manual tracking. Results depicted as mean + SEM.
